# Inked and infected: A case that highlights the emerging concern of tattoo-associated *Mycobacterium abscessus*

**DOI:** 10.1016/j.jdcr.2025.05.031

**Published:** 2025-06-19

**Authors:** Starzyk Tory, Edupuganti Neena, Celik Anastasia, Browning Jill, Banull Katherine

**Affiliations:** a Department of Dermatology, HCA Healthcare/USF Morsani College of Medicine GME, HCA Florida Largo Hospital, Largo, Florida; b Philadelphia College of Osteopathic Medicine, Suwanee, Georgia; c Bay Pines Veterans Affairs, Bay Pines, Florida

**Keywords:** atypical mycobacteria, Mycobacterium abscessus, nontuberculosis mycobacteria, rapidly growing mycobacterium, skin and soft tissue infection, tattoo complication, tattoo infection

## Introduction

In recent years, cutaneous infection with nontuberculosis mycobacteria (NTM) species has become an emerging public health concern.^1^^,^^2^ *Mycobacterium abscessus* is unique due to its highly pathogenic and multidrug-resistant nature, making this infection a therapeutic challenge.^2^^,^^3^ NTM are widespread in the environment and commonly cause cutaneous infections through penetrating skin trauma, such as in the event of a tattoo procedure.^1^^,^^4^^,^^5^ While tattoo-related *M. chelonae* infection has been frequently reported in the literature, there is limited data on *M. abscessus* infections.^1^, ^2^, ^3^

As tattoo popularity increases, it is crucial to consider the regulations surrounding safe tattoo practices and ink sterility. Although the Food and Drug Administration provides safety guidelines, local jurisdictions oversee these regulations, resulting in considerable variation across locations.^4^^,^^6^ Here, we report a case of *M. abscessus* infection following a tattoo procedure, highlighting the importance of considering NTM infections in patients with tattoo-related cutaneous eruptions that may not respond to empirical therapy.^1^^,^^2^^,^^7^

## Case report

**Fig 1:**
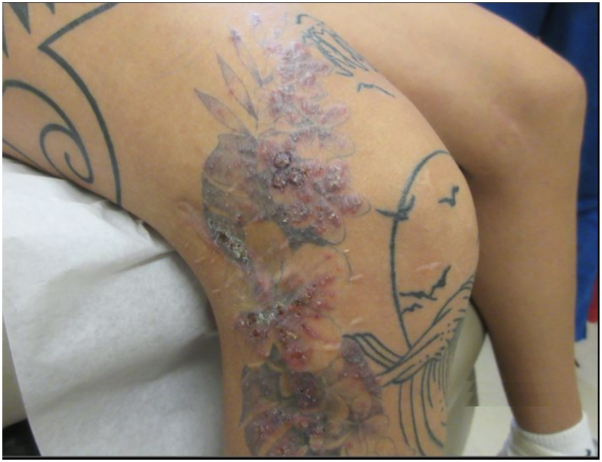
Multiple pink and purple crusted nodules and pustules in a sporotrichoid pattern localized to a tattoo on the right lower extremity.

An otherwise healthy 31-year-old female presented to a dermatology clinic in Florida to evaluate a worsening rash localized to a tattoo on her right lower extremity that she received 8 weeks before this visit (Fig 1). On exam, numerous pink and purple crusted nodules and pustules were localized to the tattoo on the right shin, knee, and thigh. The rash was tender, pruritic, non-necrotic, and spread in a sporotrichoid pattern. The patient had no recent travel, trauma, or rash with prior tattoos.

The patient initially presented to an urgent care clinic shortly after the tattoo placement for evaluation of erythema. She was placed on cephalexin, and then trimethoprim/sulfamethoxazole without improvement. Her symptoms worsened, and she was hospitalized for presumed cellulitis 3 weeks before her dermatology consultation. She was treated with intravenous vancomycin and discharged on oral doxycycline and topical mupirocin without improvement in the rash.

**Fig 2:**
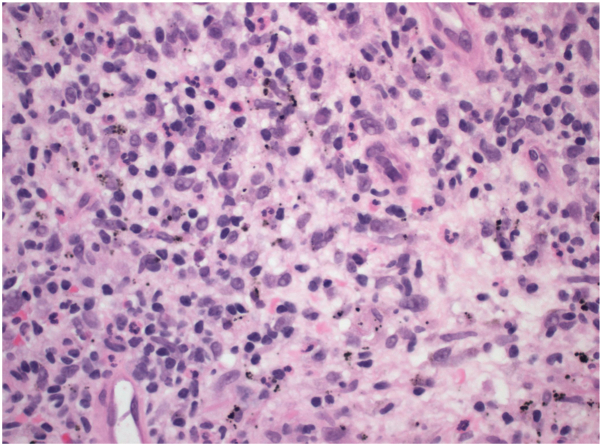
H&E−mixed inflammatory cell infiltrate (histiocytes, lymphocytes, plasma cells, neutrophils) admixed with rare giant cells and black pigment-laden macrophages in the dermis (magnification 40×). *H&E*, Hematoxylin and eosin.

**Fig 3:**
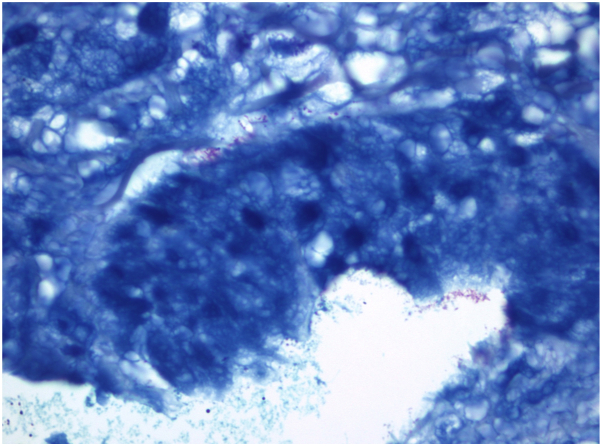
Positive Fite stain, revealing rare pink-red slender rods, consistent with mycobacteria (magnification 100×).

Two punch biopsies were performed during her initial dermatologic consultation. Hematoxylin and eosin staining revealed a suppurative, granulomatous dermatitis with associated black tattoo pigment (Fig 2). Acid-fast bacilli culture and stain, and Periodic acid-Schiff with diastase stains failed to reveal any acid-fast bacilli or fungal organisms. Fite staining was positive and revealed rare pink-red slender rods, consistent with mycobacteria (Fig 3). Fresh tissue cultures from the biopsy were inoculated into Mycobacterium Growth Indicator Tubes supplemented with Polymyxin B, Amphotericin B, Nalidixic Acid, Trimethoprim, and Azlocillin and incubated at 37 degrees Celsius. After 4 days of incubation, the BACTEC Mycobacterium Growth Indicator Tubes 960 instrument flagged the sample as positive. Subsequent strain identification was performed using polymerase chain reaction with enzyme restriction analysis targeting the *hsp65* gene, confirming the presence of *Mycobacterium abscessus*.

Drug susceptibility testing was performed using minimum inhibitory concentration measurements per Clinical & Laboratory Standards Institute and indicated azithromycin, clarithromycin, cefoxitin, and amikacin sensitivity. The organism was susceptible to clarithromycin at initial and 14-day testing. In our case, the laboratory did not provide subspecies identification; however, given the clarithromycin sensitivity, the organism was likely *M. abscessus* subsp. *massiliense* or *M. abscessus* subsp. *abscessus* C28 sequevar.

**Fig 4:**
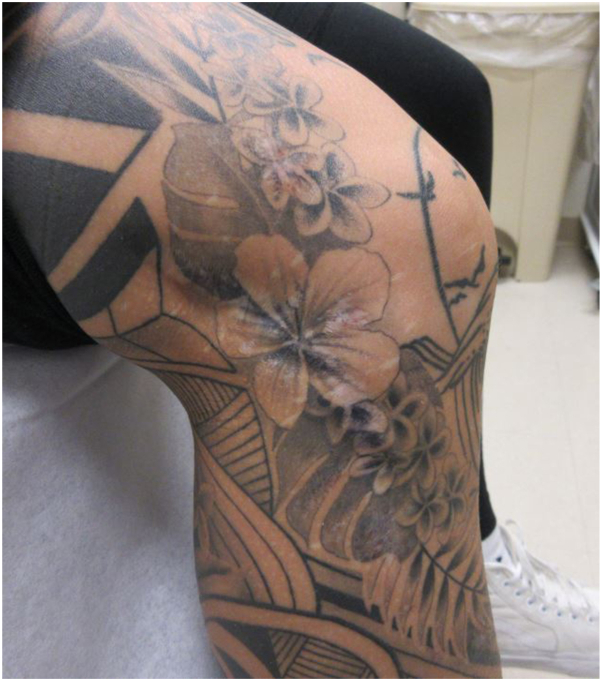
One-year follow-up visit revealed significant improvement in residual inflammation and improved appearance of scar tissue in the affected area.

The patient was referred to an infectious disease specialist, who treated her with omadacycline 300 mg daily and azithromycin 250 mg daily for 4 months. At her 6-month follow-up visit, the patient displayed clinical clearance. At her 1-year follow-up visit, residual inflammation and appearance of scar tissue significantly improved (Fig 4).

## Discussion

This case highlights a rare *M. abscessus* infection following a tattoo procedure. *M. abscessus* can cause chronic skin, soft tissue, and lung infections in both immunocompromised and otherwise healthy patients, as seen here and in several reported cases.^1^^,^^2^^,^^4^^,^^5^^,^^7^, ^8^, ^9^ Tattoo-related infections have been attributed to poor infection control practices. Commonly, infections are caused by contaminated ink or nonsterile or tap water used to dilute black ink to create shades of gray, resulting in infections localized to the gray pigment within tattoos.^2^^,^^4^^,^^8^^,^^9^

The clinical presentation of NTM infection in tattoos is nonspecific.^1^^,^^8^ Clinically, the diagnosis of tattoo-related NTM infection should be suspected in a patient with an acute eruption of papules and pustules several days to weeks following a tattoo that is refractory to treatment with empiric antibiotics, such as cephalexin or doxycycline. Punch skin biopsy with AFB culture and Fite stain should be performed to confirm infection.^4^^,^^8^ Histology reveals nonspecific dermal inflammatory infiltrates with lymphohistiocytic reactions and granulomas.^7^

Following organism identification, susceptibility testing is essential to guide antimicrobial selection. Treatment includes surgical debridement with at least 2 active agents, such as azithromycin, clofazimine, omadacycline, linezolid, or bedaquiline. If the isolate is susceptible, as in our case, azithromycin should be used due to its tolerability, safety, and efficacy profile. *M. abscessus* often displays macrolide resistance through an inducible ribosomal methylase encoded by *erm(41)*, necessitating alternative therapies.^10^ The duration of therapy is generally 4-6 months.^1^^,^^4^^,^^5^^,^^7^, ^8^, ^9^

This is the first reported case of tattoo-associated *M. abscessus* infection successfully treated with omadacycline-azithromycin combination therapy. Omadacycline, a novel aminomethylcycline antibiotic approved in 2018 for the treatment of community-acquired bacterial pneumonia and acute bacterial skin infections, has been reported to have favorable treatment outcomes against *M. abscessu*s.^10^ Omadacycline’s once-daily oral dosing and favorable side effect profile make it a promising adjunct in *M. abscessus* combination therapy.^10^

Globally, a notable rise in tattoo-associated *M. abscessus* infections has been reported. Our PubMed review identified 19 cases, 10 of which occurred in the United States. In the past decade, we identified 11 cases, 7 of which occurred in the United States.^1^, ^2^, ^3^, ^4^, ^5^, ^6^, ^7^, ^8^, ^9^ It remains unclear whether this emerging trend in cases is driven by increased tattoo popularity or improved detection and reporting of cases, a consideration worth exploring further.^5^ In conclusion, clinicians should remain vigilant in diagnosing and managing tattoo-related NTM infections.^4^^,^^6^ Furthermore, it is crucial that regulatory oversight of sterility standards amongst tattoo artists, parlors, and manufacturers be maintained.

## Conflicts of interest

None disclosed.
